# Effect of KRAS mutations and p53 expression on the postoperative prognosis of patients with colorectal cancer

**DOI:** 10.1002/mgg3.1905

**Published:** 2022-06-10

**Authors:** Lingfeng Wang, Shengtao Lin, Changshun Yang, Shaoxin Cai, Weihua Li

**Affiliations:** ^1^ Shengli Clinical Medical College of Fujian Medical University Fuzhou China; ^2^ Department of Surgical Oncology Fujian Provincial Hospital Fuzhou China

**Keywords:** colorectal adenocarcinoma, KRAS, p53, prognosis

## Abstract

**Background:**

In the occurrence and development of colorectal cancer, p53 is an important regulator downstream of the MAPK signaling pathway and plays an important role in inhibiting abnormal proliferation signals generated by KRAS mutations. The purpose of this study is to explore the correlation between KRAS mutations and p53 expression and evaluate their prognosis values in colorectal cancer.

**Methods:**

PCR technology and immunohistochemical (IHC) staining were used to detect KRAS mutation status and p53 expression level in 266 specimens of colorectal adenocarcinoma. Based on p53 expression level, these were divided into high expression and normal groups. Patients with KRAS mutations were divided into mutant and wild‐type groups. The two were combined with each other to analyze the relationship between patients' clinical data and their impact on the prognosis.

**Results:**

KRAS mutations were found in 38.6% of the patients and 40.8% had a high p53 expression. There was no significant difference in the overall survival rate of patients, with or without KRAS gene mutations, and p53 expression level. In the group of patients with KRAS mutations, the survival time of patients with a high p53 expression was significantly lower than that of patients with a normal p53 expression (*p* = 0.020, log‐rank test). Multivariate analysis showed that p53 high expression is an independent risk factor for the overall survival time of patients with KRAS mutations (HR = 2.330, 95% CI = 1.041–5.216, *p* < 0.05).

**Conclusion:**

Colorectal cancer patients with KRAS mutations with a high p53 expression have a poor prognosis.

## Graphical abstract

The purpose of this study is to explore the correlation between KRAS mutations and p53 expression and evaluate their prognosis values in colorectal cancer. PCR technology and immunohistochemical (IHC) staining were used to detect KRAS mutation status and p53 expression level in 266 specimens of colorectal adenocarcinoma. Finally, we found colorectal cancer patients with KRAS mutations with a high p53 expression have a poor prognosis.

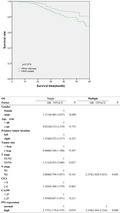

## 1 INTRODUCTION

Colorectal cancer (CRC) is one of the most common cancers worldwide that is responsible for serious damage to human health, and a reduction in the survival of affected patients. Colorectal adenocarcinoma accounts for approximately three‐quarters of colorectal cancer cases (Jemal et al., 2008). Although considerable progress has been made in CRC pathogenesis and clinical treatment, tumor resection is still the preferred treatment, and the 5‐year survival rate is less than 65% (Siegel et al., 2017). Therefore, identifying relevant signs of poor prognosis and investigating CRC progression, are high priorities for researchers and clinicians.

The traditional pathogenic pathway, the adenoma–carcinoma sequence, plays an important role in the carcinogenesis of most colorectal cancers (De Sousa et al., 2013). APC, KRAS, DCC, TP53, and DNA mismatch repair (MMR) are some of the genes that are associated with the adenocarcinoma pathway. Among these, KRAS mutations occur in the intermediate and advanced stages of CRC, and TP53 mutations occur in the advanced stages (Vogelstein et al., 1988).

The p53 gene is an important tumor suppressor that is stimulated by DNA damage, oxidative stress, and activated oncogenes to produce p53 protein, which induces DNA repair, apoptosis, and controls cell cycle checkpoints. TP53 mutations lead to the loss of its tumor suppressor function and increase tumor invasiveness and metastasis resulting in reduced survival rates (Dittmer et al., 1993; Dong et al., 2007; Willis et al., 2004). The p53 protein is difficult to detect in cells, and the mutant p53 protein is poorly hydrolyzed. Various methods can be used to detect p53 mutant protein, including immunohistochemistry (IHC) methods (Willis et al., 2004).

The KRAS gene is located on the short arm of chromosome 12 and encodes a 21‐kDa signaling protein that activates the transduction of the MAPK and PI3K/AKT pathways (Banno et al., 2014). RAS is inactive when combined with GDP, but growth factors, such as EGF and PDGF, stimulate the GDP and GTP cellular exchange, leading to conformational changes that produce active RAS, which stimulate downstream factors involved in cell proliferation. In normal cell environment, the protein has a GTPase activity and can inactivate the KRAS gene after signal transduction. The mutant KRAS can remain active despite a limited EGFR stimulation. The GTPase activity of the mutant gene is lower than that of the wild‐type KRAS, resulting in a 3–9 times reduction of its hydrolytic activity compared to that of the wild‐type KRAS. This anomaly results in irreversible signals that lead to uncontrollable cell proliferation and differentiation (Hunter et al., 2015; Tan & Du, 2012). Previous studies reported that p53 is an important regulatory factor, downstream of the MAPK signaling pathway that can induce apoptosis and maintain the normal progression of the cell cycle (Peltomäki, 2012), while the loss of p53 function is conducive to cell proliferation.

The expression of KRAS and p53 genes are mostly related to the pathological characteristics and treatment response of CRC patients, and therefore, are widely used to predict the outcomes of CRC treatments (Al‐Kuraya, 2009; Chaar et al., 2014; Ross, 2012). However, few studies have reported the influence of the interaction between KRAS and p53 gene on the prognosis of patients with colorectal adenocarcinoma. To solve these issues, we determined the KRAS mutation status and p53 expression level in patients with colorectal adenocarcinoma and evaluated their influence on the prognosis of patients.

## 2 MATERIALS AND METHODS

### 2.1 Patients

From June 2012 to September 2015, 266 stage III (according to the staging of the Union for International Cancer Control) colorectal cancer surgical specimens were collected from the Fujian provincial hospital (Fuzhou, China). More than two deputy chief physicians of the pathology department confirmed that they were primary colorectal adenocarcinoma. To unify the treatment, we did not include advanced colorectal carcinoma. All CRC patients were sporadic cases and none of the patients who were included in the study had BRAF mutations, mucinous/signet ring cell carcinoma, or secondary primary tumors. Before surgery, none of the patients received radiotherapy, chemotherapy, or other drugs, and all primary lesions were surgically removed. Postoperative chemotherapy was performed with oral Xeloda and intravenous systemic chemotherapy (XELOX). We recorded the patients' primary tumor characteristics, including the primary tumor site, the tumor size (the largest primary lesion from multiple tumors was taken as an indicative lesion), T stage, lymph node status, p53 expression levels, KRAS mutation status, CEA level in the serum, the level of the tumor biomarker CA199, and the patients' survival time.

### 2.2 PCR detection of KRAS mutations

Three tissue sections, with a thickness of 5 μm, were placed into a 1.5‐ml EP tube and deparaffinized with xylene. Genomic DNA was extracted using a DNA isolation kit (AmoyDx, Xiamen Diagnostics Co., Ltd.). To determine the purity of the DNA, the A260/A280 ratio of absorbance of the DNA sample was set between 1.8 and 2.1 using the Nanodrop 2000 spectrophotometer. According to the manufacturer's instructions, the AmoyDx® KRAS mutation detection kit (Amoy Diagnostics Co., Ltd.) was used to determine KRAS mutation status in each DNA sample, and a scorpion amplification refractory mutation system (Amoy Diagnostics Co., Ltd) was used to detect seven KRAS mutation sites in codons 12 and 13. The PCR reaction parameters are: 42 °C 5 min and 95 °C 5 min, 1 cycle; 95 °C 25 s, 64 °C 20 s, 72 °C 20 s, a total 10 cycles; 93 °C 20 s, 60 °C 35 s, 72 °C 20 s, a total of 30 cycles. The third stage collected signals at 60 °C (Li et al., 2019).

### 2.3 Immunohistochemistry (IHC) detection of p53 protein expression

The mouse antihuman p53 (DO‐7), the citric acid antigen recovery solution (pH = 6.0), the two‐step Elivision TM plus (KIT‐9903) immunohistochemical detection kit, and the phosphate buffer PBS and DAB color reagent kit (DAB‐0031) were purchased from Fuzhou Maixin Biotechnology Development Co., Ltd. According to the product instructions, the protein expression was detected by the two‐step immunohistochemical Elivision method. The microwave antigen retrieval was performed before staining and DAB was used for color development. PBS, instead of the primary antibody, was used as a negative control. The criterion for p53 positive staining results is the presence of yellow or brown or tan particles in the nucleus. No positive cells or positive cells <5% are negative, positive cells 5%–25% are weakly positive (+), 25% ~75% is positive (++), >75% is strong positive (+++). We refer to patients with p53 staining greater than 75% as the p53 high‐expression group, and the rest as the normal group.

### 2.4 Postoperative follow‐up

We recorded the overall survival time of all patients and OS was defined as the time from the surgery of the patient to death from any cause. The follow‐ups were conducted by telephone and outpatient reexamination. The patients received combined tomography, approximately every 3 months, and more frequently for patients with clinical suspicion of progression. The last follow‐ups occurred in February 2019.

### 2.5 Statistical analysis

The SPSS20.0 statistical software package was used for statistical testing of all data. The chi‐square or corrected chi‐square test was used to analyze the counting data, and the exact Fisher probability method was used on small data samples. The Kaplan–Meier method and log‐rank test were used for survival analysis. The Cox proportional hazards regression model was used to determine the hazard ratio (HR) for the 95% confidence interval (CI). *p* < 0.05 was considered to be a significant difference.

## 3 RESULTS

### 3.1 Clinicopathological features

The study included 154 males (55.6%) and 112 females (40.4%) with an age that ranged from 23 to 87 years and an average age of 63 years. There were 159 cases (57.4%) of KRAS wild type and 107 cases (38.6%) of KRAS mutant type. There were 113 cases (40.8%) with a high p53 expression, and 153 cases (55.2%) with a normal expression (Table 1).

**TABLE 1:** Patient's clinical characteristics

Factor	Number
Total number	266
Age, years (range)	63 (23–87)
Gender (male/female)	154/112
Primary tumor location	
Right side	81
Left side	185
Tumor size	
<5 cm	167
≥5 cm	99
T stage	
T1/T2	123
T3/T4	143
N stage	
N1	199
N2	67
CEA (ng/ml)	
<5	131
≥5	135
CA19‐9 (U/ml)	
<27	182
≥27	84
KRAS	
Mutations	107
Wild	159
p53 protein	
High expression	113
Normal	153

Table 2 shows the relationship between p53 expression level and the clinicopathological factors in the KRAS state of CRC patients. Among them, 43 patients (16.2%) with KRAS mutations had a high p53 expression and 70 of patients (26.3%) were categorized in the wild‐type KRAS group. There was no significant difference among those factors.

**TABLE 2:** The relationship between p53 expression and the clinicopathological factors in CRC patients

	wtKRAS			mKRAS		
P53 expression	High	Normal	*p* value	High	Normal	*p* value
Factor						
Gender						
Male	45	48	0.188	22	39	0.317
Female	25	41		21	25	
Age, year						
<60	27	28	0.349	19	20	0.173
≥60	43	61		24	44	
Primary tumor location						
Right	16	27	0.299	16	22	0.764
Left	54	62		27	42	
Tumor size						
<5 cm	46	51	0.280	26	44	0.377
≥5 cm	24	38		17	20	
T stage						
T1/T2	38	40	0.242	17	28	0.665
T3/T4	32	49		26	36	
N stage						
N1	56	75	0.483	24	44	0.173
N2	14	14		19	20	
CEA						
<5	42	44	0.185	18	27	0.973
≥5	28	45		25	37	
CA199						
<27	54	61	0.229	25	42	0.433
≥27	16	28		18	22	

### 3.2 Overall survival

**FIGURE 1:**
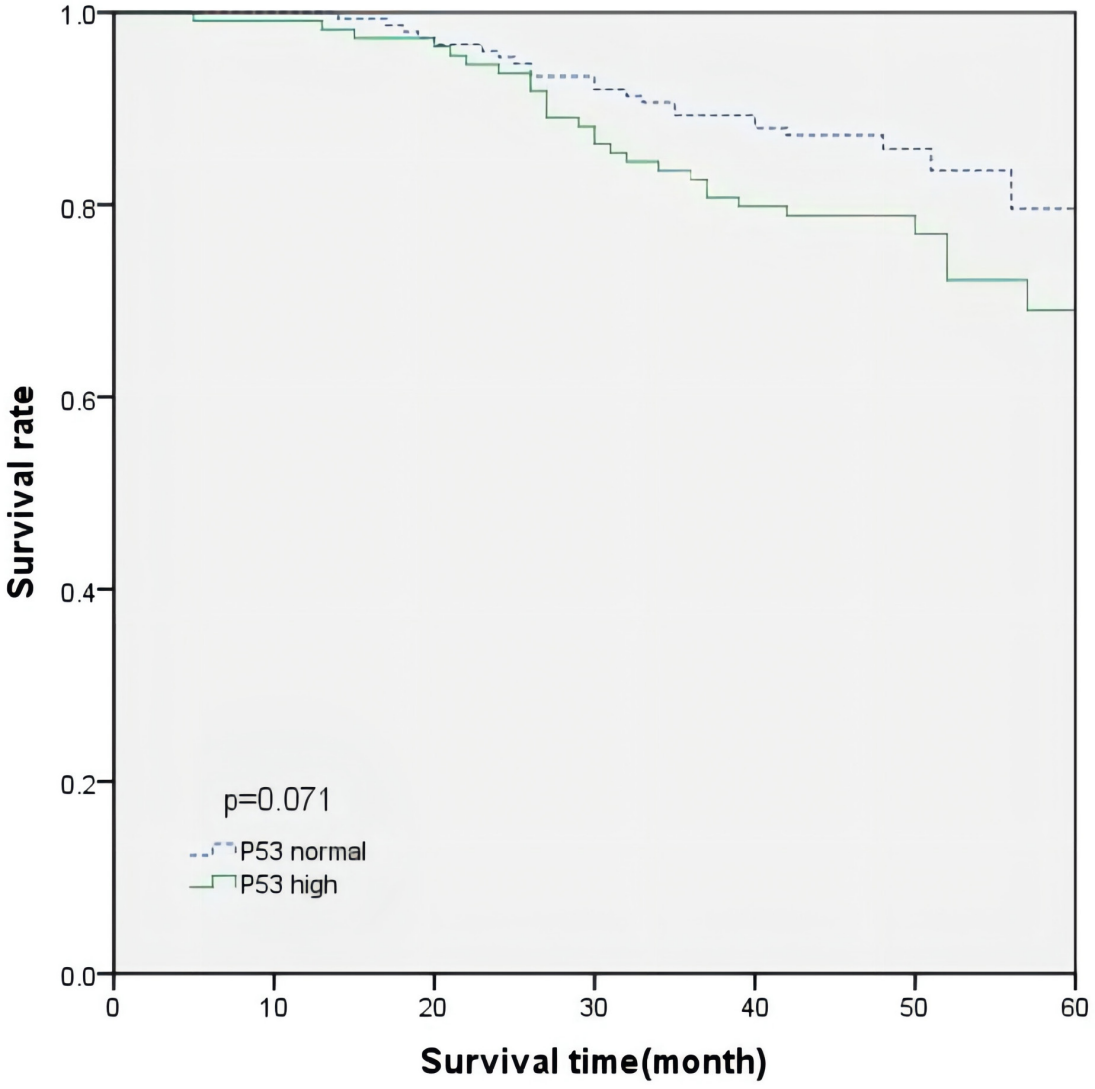
OS curve in patients according to p53 expression levels

**FIGURE 2:**
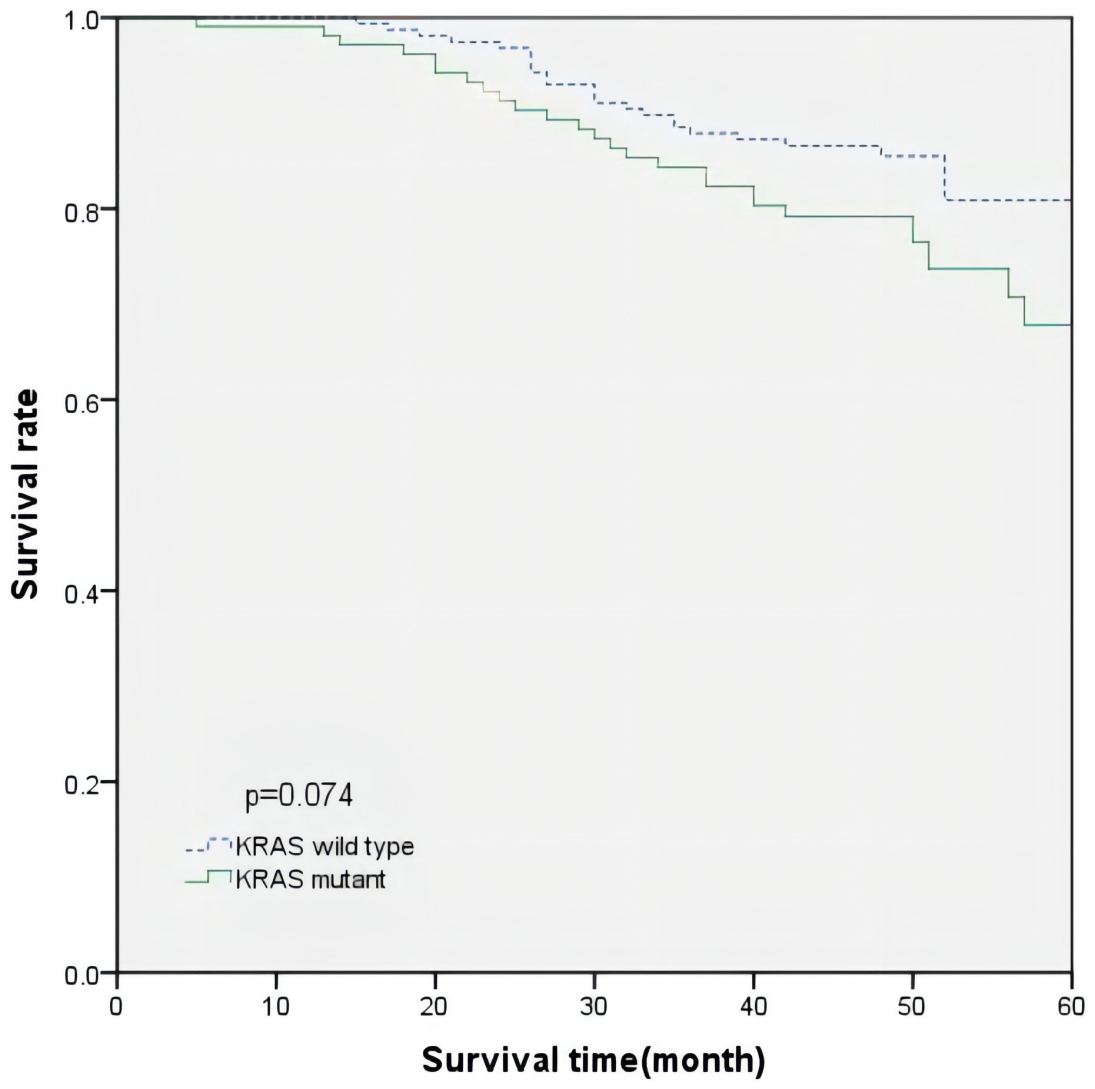
OS curve in patients according to KRAS status

**FIGURE 3:**
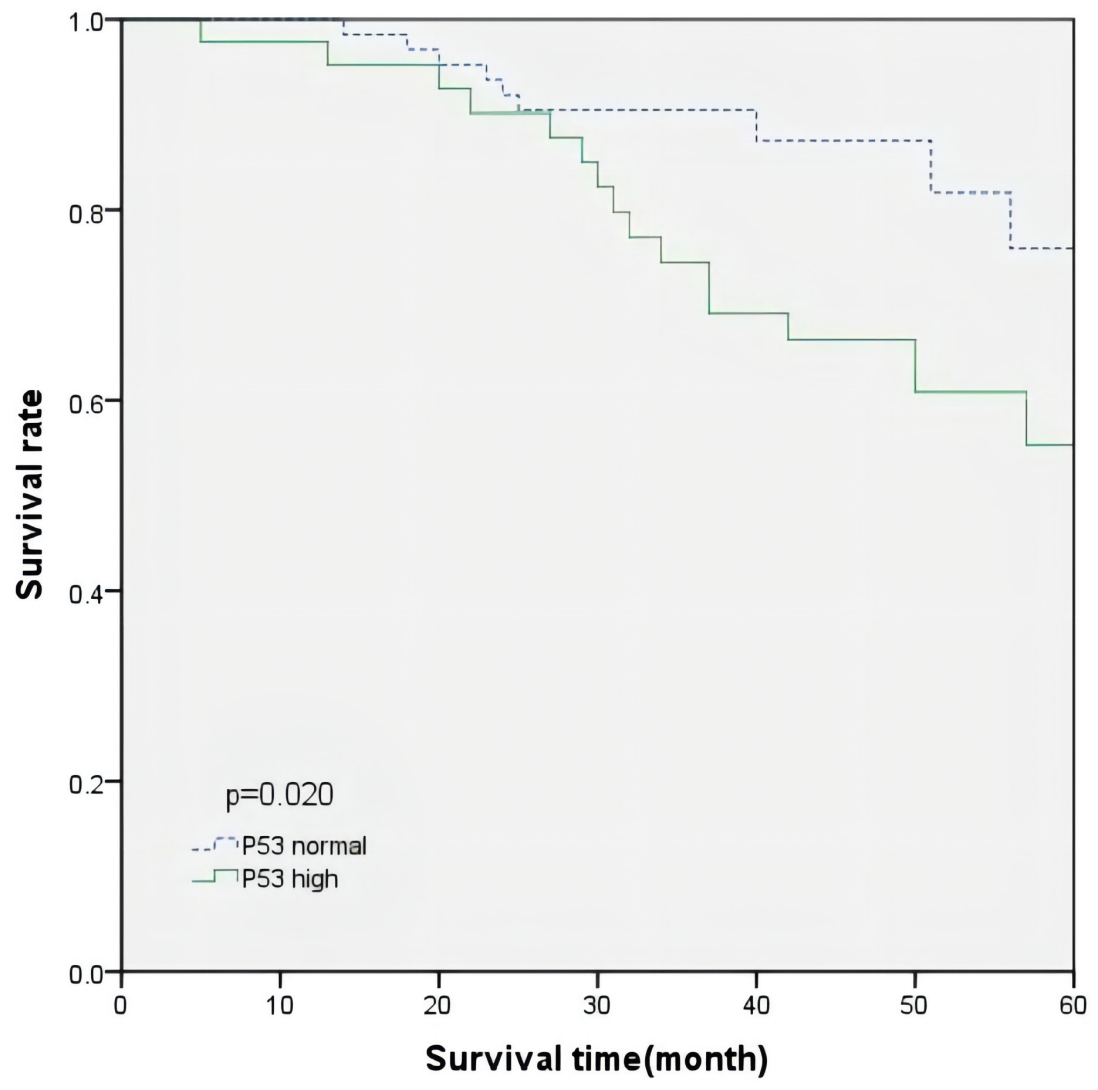
OS curve in KRAS‐mutated patients according to p53 expression levels

The overall survival time of all patients averaged 68.3 months. The Kaplan– Meier curve of OS time for all patients is shown in Figures 1 and 2. According to KRAS status and p53 expression levels, the average OS time of patients with a normal p53 expression level was 70.5 months, and the average OS time of patients with a high p53 expression was 65.6 months. The difference was not statistically significant (*p* = 0.071, log‐rank test). Similarly, the average OS of patients with a KRAS mutation was 64.6 months, and that of patients with the wild‐type KRAS was 70.6 months; however, the difference was not statistically significant (*p* = 0.074, log‐rank test). We further explored the effect of p53 expression level on the prognosis of patients based on KRAS status, and the p53 expression level in patients with wild‐type KRAS that had no significant prognostic difference. In patients with KRAS mutations, a higher p53 expression was significantly associated with worse survival (*p* = 0.020, log‐rank test) (Figure 3).

The Cox proportional hazards regression model was used to analyze the risk factors of patients with KRAS mutations (Table 3). In the univariate analysis, the high‐expression level of p53, was significantly related to lower OS. In the multivariate analysis, the high p53 expression was an independent risk factor of OS in patients with KRAS mutations (HR 2.330, 95% CI, 1.041–5.216 log‐rank test, *p* < 0.05). In addition, the status of lymph node metastasis was also an independent risk factor for the prognosis of patients with KRAS mutations (HR 2.274, 95% CI, 1.028–5.031 log‐rank test, *p* < 0.05).

**TABLE 3:** The Cox risk analysis of OS in patients with KRAS mutations

OS	Single		Multiple	
Factor	HR (95% CI)	*p*	HR (95% CI)	*p*
Gender				
Female	1			
Male	1.213 (0.485–3.037)	0.680		
Age, year				
<60	1			
≥60	0.853 (0.313–2.319)	0.755		
Primary tumor location				
Left	1			
Right	1.334 (0.553–3.217)	0.521		
Tumor size				
<5 cm	1			
≥5 cm	0.466 (0.184–1.180)	0.107		
T stage				
T1/T2	1			
T3/T4	1.111 (0.433–2.848)	0.827		
N stage				
N1	1			
N2	2.004 (0.794–5.057)	0.141	2.274 (1.028–5.031)	0.043
CEA				
<5	1			
≥5	1.103 (0.360–3.379)	0.863		
CA199				
<27	1			
≥27	1.939 (0.687–5.471)	0.211		
P53 expression				
Normal	1		1	
High	2.737 (1.179–6.355)	0.019	2.330 (1.041–5.216)	0.040

## 4 DISCUSSION

In our retrospective cohort study, KRAS status and p53 expression level had no significant effect on the prognosis of patients with colorectal cancer. The log‐rank test results showed that in patients with KRAS mutations, the overall survival time of the p53 high expression and the normal groups are significantly different. After adjusting for other possible confounding factors using multivariate analysis, a high p53 expression in tumor tissues was found to be an independent predictor of a short overall survival of CRC patients with KRAS mutations. Therefore, the results of this study suggest that a high p53 expression in tumor tissues is a good biomarker of poor prognosis in KRAS‐mutated CRC patients.

Mutations in the tumor suppressor gene p53 may result in a loss of its normal activity, leading to the inhibition of cell apoptosis, the promotion of the malignant transformation of cells, and the enhancement of invasiveness (Cooks et al., 2013). One of the most known p53 interacting protein is double minutes 2 (Mdm2) that was discovered in 1992. This protein binds and covers the p53 transactivation domain which prevents p53 function. It can also act as an E3 ubiquitin ligase, which causes p53 degradation by the intracellular ubiquitin pathway. The interaction between p53 and mdm2 is a negative feedback loop, which inhibits the abnormal activation of p53 (Park et al., 2016). The clinical significance of p53 in colorectal cancer is still controversial and some studies have shown that p53 mutations were not significantly related to the prognosis of CRC patients (Osumi et al., 2015; Tollenaar et al., 1998). In another study, p53 gene alterations were regarded as negative predictors of local disease control and metastasis (Ito et al., 2003).

It is well known that KRAS is located downstream of the EGFR signaling pathway, where it affects cell migration, adhesion, proliferation, differentiation, and apoptosis. Furthermore, KRAS is associated with tumor formation and malignant transformation. KRAS mutations lead to the production of abnormal and permanently active KRAS proteins, leading to constitutive intracellular signaling, uncontrolled cell proliferation, and cancer (Arrington et al., 2012). In KRAS wild‐type patients, KRAS is always active due to EGF continuous stimulation. Patients with wild‐type KRAS respond better to drugs, such as cetuximab and panitumumab (antiepidermal growth factor receptor); However, KRAS mutants do not respond to these drugs (Van Cutsem et al., 2011). Therefore, KRAS mutation status is commonly used in clinic to predict whether the patient's anti‐EGFR treatment is effective. In this study, KRAS mutation rate was ~40%, which is similar to that of previous reports (Downward, 2003). Moreover, KRAS mutations have not been related to the prognosis of CRC patients.

Previous studies have shown that p53 is closely related to the EGFR signaling pathway, and the activation of the EGFR signaling pathway can regulate the expression of p53 protein (Wu, 2004). Meanwhile, the mutant p53 proteins can enhance the transmission of EGFR signal, promote ERK1/2 phosphorylation, and activate the MAPK pathway, which promote cell proliferation and differentiation, invasion, and epithelial–mesenchymal transition and other processes (Muller et al., 2013; Sauer et al., 2010).

TP53 and KRAS seem to be related to each other in adenoma cancer. However, few studies have investigated their relationship in CRC patients. A study by the University of Texas showed that if patients with colorectal cancer liver metastasis have both TP53 and KRAS mutations, the overall survival rate would be significantly reduced (Chun et al., 2019). Research by Garcia‐Aguilar et al. (2011) also confirmed that a combination of KRAS and TP53 mutations can enhance the resistance of patients with advanced CRC to neoadjuvant chemotherapy. However, Daitoku et al., (2020) reported that a high p53 expression cannot be used as an independent prognostic factor for patients with liver metastases that are originated from colon cancer with KRAS mutations.

Unlike the previous studies, our research focused on CRC patients who are not affected by liver metastasis. This study shows that a high level of p53 expression is an independent predictor of a shortened overall survival of CRC patients with KRAS mutations. Some in vitro experimental results can explain the above phenomenon: (1) The occurrence of KRAS mutations may cause excessive cell proliferation; (2) This hyperproliferation signal is a stress signal, which can trigger the release of wild p53 from mice mdm2; (3) the released wild p53 can stop the cell cycle, initiate the apoptosis program, and protect the cells from abnormal growth factors (Suh et al., 2011); and (4) The mutant p53 lost its original tumor suppressive function and cooperated with the KRAS mutant gene to promote cancer progression (McMurray et al., 2008). Therefore, for patients with KRAS mutations, if there is a p53 mutation, it means that the prognosis is worse than that of patients with wild‐type p53.

This study has several limitations. First, it is a retrospective study, and it was performed in a separate institution, and therefore, there may have a selection bias. Second, the false positive rate of p53 protein expression in healthy subjects is ~5%, which may cause the p53 protein partially reflect the tumor's TP53 mutation (Luo et al., 1995). Thirdly, unlike some previous studies, KRAS mutation status and p53 expression levels had no significant effects on the prognosis of all patients. This may be due to the patients' different stages of tumor progression and treatment methods.

In summary, p53 high‐expression level can be used as an independent risk and prognostic factor for patients with KRAS mutations, which is helpful in judging the malignant degree, progression, and postoperative survival of the tumor. In this study, the selected sample size and molecular indicators were relatively small, and it is only through large sample sizes, and multicenter, multi‐index, and prospective studies that we can obtain more accurate and comprehensive evaluation in the future.

## AUTHOR CONTRIBUTIONS

Lingfeng Wang: Methodology, Software, Writing‐ Original draft preparation. Shengtao Lin: Validation, Investigation. Changshun Yang: Supervision, Visualization. Shaoxin Cai: Formal analysis, Data Curation. Weihua Li: Conceptualization, Writing ‐ Review & Editing.

## CONFLICT OF INTEREST

The authors have declared that no competing interests exist.

## ETHICAL COMPLIANCE

The study was conducted in accordance with the Declaration of Helsinki (as revised in 2013). The protocol was approved by the institutional review boards of Fujian Provincial Hospital (no. K2018‐08‐033), and informed consent was obtained from all patients before inclusion in the study.

## Data Availability

The data that support the findings of this study are available on request from the corresponding author. The data are not publicly available due to privacy or ethical restrictions.
